# Elevated levels of cell-free NKG2D-ligands modulate NKG2D surface expression and compromise NK cell function in severe COVID-19 disease

**DOI:** 10.3389/fimmu.2024.1273942

**Published:** 2024-02-12

**Authors:** Daniel Fernández-Soto, Álvaro F. García-Jiménez, José M. Casasnovas, Mar Valés-Gómez, Hugh T. Reyburn

**Affiliations:** ^1^ Department of Immunology and Oncology, National Centre for Biotechnology (CNB), Spanish National Research Council (CSIC), Madrid, Spain; ^2^ Department of Macromolecular Structures, National Centre for Biotechnology (CNB), Spanish National Research Council (CSIC), Madrid, Spain

**Keywords:** COVID-19, natural killer cells, ADCC - antibody dependent cellular cytotoxicity, NKG2D (natural killer group 2 member D), soluble NKG2D ligands

## Abstract

**Introduction:**

It is now clear that coronavirus disease 19 (COVID-19) severity is associated with a dysregulated immune response, but the relative contributions of different immune cells is still not fully understood. SARS CoV-2 infection triggers marked changes in NK cell populations, but there are contradictory reports as to whether these effector lymphocytes play a protective or pathogenic role in immunity to SARS-CoV-2.

**Methods:**

To address this question we have analysed differences in the phenotype and function of NK cells in SARS-CoV-2 infected individuals who developed either very mild, or life-threatening COVID-19 disease.

**Results:**

Although NK cells from patients with severe disease appeared more activated and the frequency of adaptive NK cells was increased, they were less potent mediators of ADCC than NK cells from patients with mild disease. Further analysis of peripheral blood NK cells in these patients revealed that a population of NK cells that had lost expression of the activating receptor NKG2D were a feature of patients with severe disease and this correlated with elevated levels of cell free NKG2D ligands, especially ULBP2 and ULBP3 in the plasma of critically ill patients. In vitro, culture in NKG2DL containing patient sera reduced the ADCC function of healthy donor NK cells and this could be blocked by NKG2DL-specific antibodies.

**Discussion:**

These observations of reduced NK function in severe disease are consistent with the hypothesis that defects in immune surveillance by NK cells permit higher levels of viral replication, rather than that aberrant NK cell function contributes to immune system dysregulation and immunopathogenicity.

## Introduction

For the majority of people infected with the Severe acute respiratory syndrome coronavirus 2 (SARS-CoV-2), the evolution of the infection is either asymptomatic or they develop only mild symptoms of disease (1–4). In contrast, some individuals infected with this virus become critically ill and a significant fraction of these patients die. Multiple factors, including age and comorbidities such as diabetes, obesity, and pre-existing cardiovascular or pulmonary disease influence the severity and mortality of COVID-19 (5, 6). However, variations in the immune response to SARS-CoV-2 can also influence the clinical evolution after infection. Indeed, it appears that an immunopathogenic, dysregulated immune response, including cytokine storms, is one of the major contributory factors leading to multi-organ failure and death. It has been shown that some individuals are impaired in their ability to mount an effective immune response due to genetic lesions, that often affect Type I Interferon pathways (7–9). However, for the majority of patients who develop life-threatening disease, the basis of the defective immune response underlying their failure to adequately control virus replication is not well understood. Multiple aspects of adaptive immunity to SARS-CoV-2 have been studied intensively, and here we analyse the phenotype and function of Natural Killer (NK) cells in patients with severe or mild COVID-19 disease.

NK cells are innate effector lymphocytes activated during ongoing viral infection (10–13), that help clear virus-infected cells through multiple mechanisms, including direct cytotoxicity of infected cells as well as cytokine or chemokine secretion. NK cells can also indirectly influence the development of downstream immune responses via crosstalk with dendritic cells and T cells (14–16). Clear evidence for a relevant role of NK cells in protection against viral infections comes from patients with selective NK cell deficiencies, who often suffer fulminant viral infections, predominantly with herpes viruses (17, 18). Since a rapid NK cell response is also a feature of the acute phase of infections in humans with a range of RNA viruses (19–23), it is plausible to suggest that NK cells could act early to limit viral replication in the early stages of infection with SARS-CoV-2, but that defects in the NK response could contribute to the development of severe COVID-19 disease (24, 25). In fact, a number of studies have reported that patients with severe disease have low numbers of peripheral blood T and NK cells (26–30), whereas increased numbers of NK cells may be found in the infected lung. Increased numbers of NK cells expressing markers of activation are found in COVID-19 patients, however the ability of these activated circulating NK cells to mediate natural cytotoxicity is reduced (31). Adaptive NK cells (CD57^hi^, NKG2C^+^, FcϵR1γ^-^, CD85j^+^), that are more specialised to mediate antibody dependent cellular cytotoxicity (ADCC), are also more frequent in patients with severe disease (31–33) as are a population of TNF-α producing NK cells with an activated memory-like phenotype are a feature of patients with life-threatening COVID-19 disease (34). It has been reported that NK subsets with high DNAM1 expression were increased in patients who cleared virus more rapidly (35). However, it should be pointed out that viral loads in these patients were defined by qRT-PCR analysis of serial nasal swabs and whether Ct values from SARS-CoV-2 diagnostic PCR assays are reliable indicators of viral load is controversial (36, 37). Finally, it has been reported that *in vitro*, NK cells can kill SARS-CoV-2 infected cells to control virus replication and that viral load decline in COVID-19 correlates with NK cell status (38). Moreover, in primate models of SARS-CoV-2 infection, viral persistence in lung alveolar macrophages has been reported to be controlled by IFN-γ and NK cells (39). Thus, different aspects of NK cell responses have been associated with the severity of COVID-19 disease, but the factors regulating NK activation state in COVID-19 are still not clearly understood.

Here we present detailed analyses comparing the phenotype and function of peripheral blood NK cells of SARS-CoV-2 infected patients who developed life-threatening disease with those of SARS-CoV-2 infected individuals with only very mild symptoms of infection. We could confirm higher levels of adaptive NK cells and activated peripheral blood NK cells in critically ill COVID-19 patients, and we also show that the NK cells of these patients are less competent functionally than NK cells from patients with mild/asymptomatic disease. Specifically, NK cells from critically ill patients were less able to mediate ADCC and expressed lower levels of the activating receptor NKG2D. Increased levels of the NKG2D ligands (NKG2DL) ULBP2 and ULBP3 were detected in plasma from patients with life-threatening COVID-19 disease. *In vitro*, culture in sera from patients with severe disease led to reduced ADCC function of healthy donor NK cells that could be blocked by the addition of NKG2DL-specific antibodies.

## Materials and methods

### Study subjects

Samples used in this study were provided by the Biobank Hospital Universitario Puerta de Hierro Majadahonda (HUPHM)/Instituto de Investigación Sanitaria Puerta de Hierro-Segovia de Arana (IDIPHISA) (PT17/0015/0020 in the Spanish National Biobanks Network). The use of these samples was approved by the Research Ethics Committee of the CSIC (173/2020) and the biobank (AC0099-A-2021). Experiments were carried out following the ethical principles established in the Declaration of Helsinki. Clinical information about these individuals has been published previously (40). Briefly, outpatient samples were from individuals who presented mild symptoms of COVID-19, tested positive in PCR, but recovered spontaneously without hospitalisation or further medical intervention. In contrast, the samples defined as critical corresponded to patients who developed severe COVID-19 disease that required admission to intensive care and mechanically assisted ventilation. The major features of the clinical presentation of these severely ill patients have been described previously (40). The majority of these patients presented in March/April 2020, and were treated with Tocilizumab, Ritonavir, Lopinavir and β-lactam antibiotics as well as prophylactic therapy with heparin (1.5mg/kg/day). The healthy control samples were collected prior to the COVID-19 pandemia and were also obtained from the HUPHM/IDIPHISA biobank. Further information on the age/sex composition of the patient groups, including timings of when samples from these different groups of patients were collected, is summarised in **Supplementary Tables 1**, **2**.

### Sample preparation and storage

Whole blood was collected in acid citrate dextrose (ACD)-containing Vacutainers and processed on the day of collection. Peripheral blood mononuclear cells (PBMCs) were isolated by density centrifugation over Ficoll-Paque media and frozen in 90% foetal bovine serum/10% dimethyl sulfoxide [DMSO]). Cells were frozen to −80°C in a controlled-rate freezing chamber before transfer to liquid nitrogen for long-term storage.

### PBMC isolation and culture

Cryopreserved PBMCs were thawed in warmed RPMI 10% FBS and incubated with 25 U/ml Benzonase (Merck #70746) for 10minutes at RT. After two washes, the cells were incubated overnight in RPMI 10% FBS to recover and used in NK activation experiments the next day.

### ELISA assays

Antibody responses to a range of SARS-CoV-2 antigens were characterised using a previously described protocol (40). Levels of MICA/B, ULBP1, ULBP2/5/6 and ULBP3 in plasma were assayed as previously described (41) and the antibodies used in these experiments are detailed in **Supplementary Table 3**. The levels of plasma TGFβ were measured using the Human TGF-beta 1 DuoSet ELISA from R&D Systems. MICA-specific autoantibodies were assayed by ELISA using recombinant MICA protein (Sinobiological).

### ELISA-based NK activation assay

This assay was based on that described by Chung et al. (42). Briefly, purified, recombinant Spike protein (43) was plated overnight in Thermo NUNC MaxiSorp plates at 3 μg/ml in BBS at 4°C. After 3 washes with PBS-T, wells were blocked overnight in PBS 1% Casein (PBS-C) at 4°C. Then sera were added in a 1:50 dilution in PBS-C and incubated at RT for 2 hours. After 3 washes in PBS-T and 3 more in PBS, 50-100,000 PBMC were added per well in 100μl of RPMI medium containing 10% FBS, 5 μg/ml Brefeldin A (Biolegend #420601) and 1 μg/ml anti-LAMP1-APC antibody. After a 5-hour incubation at 37°C and 5% CO_2_, cells were transferred to a U-bottom 96-well plate and stained with anti-CD3-FITC and anti-CD56-PC5 antibodies in PBA for 30’. After a 5-minutes fixation in 4% PFA, cells were incubated with anti-MIP1β-PE antibody in 0.25% Saponin. Stained cells were analysed using a Cytoflex flow cytometer (Beckman Coulter).

### Flow cytometry

Unstimulated PBMCs were stained after thawing from storage in liquid nitrogen. For immunofluorescence staining, PBMCs were washed with PBS containing 1% FBS, 0.5% BSA, and 0.05% sodium azide (PBA). Subsequently, PBMC were stained with directly labelled antibodies (**Supplementary Table 3**) and incubated for 40 minutes in the dark at 4˚C. After washing, the samples were analysed using a CytoFLEX flow cytometer. Flow cytometry data were analysed using CytExpert or Kaluza software (both from Beckman Coulter).

### CITRUS analysis

NK cell populations were identified by gating on CD3^-^CD56^+^ lymphocytes and the NK cell data were then exported for analysis using the CITRUS (cluster identification, characterisation, and regression) algorithm (44) using the Cytobank platform. Subjects with too few NK cells were excluded from analysis. Cluster sizes were limited to a minimum of 5% of total cells and only models where the false discovery rate (fdr) was below 1% were analysed further.

### Degranulation assays

150.000 PBMCs from healthy donors were incubated 18 h in 10% sera (either from COVID-19 patients or healthy controls) in RPMI 10% FBS in the presence or absence of a 5 μg/ml mix of anti-NKG2D ligands antibodies (anti-MICA/B, anti-ULBP2, anti-ULBP3). After that, cells were incubated for 2 hours with P815 cells pre-incubated with 10 μg/ml of anti-CD16 antibody to stimulate re-directed lysis via this receptor. Then, LAMP1 surface expression was evaluated by flow cytometry as previously described (45).

### Analysis of RNA-seq data

The expression of different NKG2D ligands was analysed using publicly available, pre-processed RNA-seq data (NCBI GEO database, GSE150316) obtained by RNASeq analysis of RNA extracted from FFPE slides of thin lung tissue sections prepared from autopsy specimens from patients who died after developing either severe COVID-19 disease or non-COVID pneumonia (46). Briefly, samples were filtered to analyse only those coming from lungs. Normalisation was performed using the DESeq2 R package, and different lung RNA-seq runs coming from the same sample were considered replicates and were grouped together using the collapseReplicates function. To study the expression of NKG2DL-related genes, SARS-CoV-2 infected and non-infected lungs were compared using the Log2 normalised counts (plus pseudocount). R packages ggplot2 and pheatmap were used to visualise the data.

## Results

**Figure 1:**
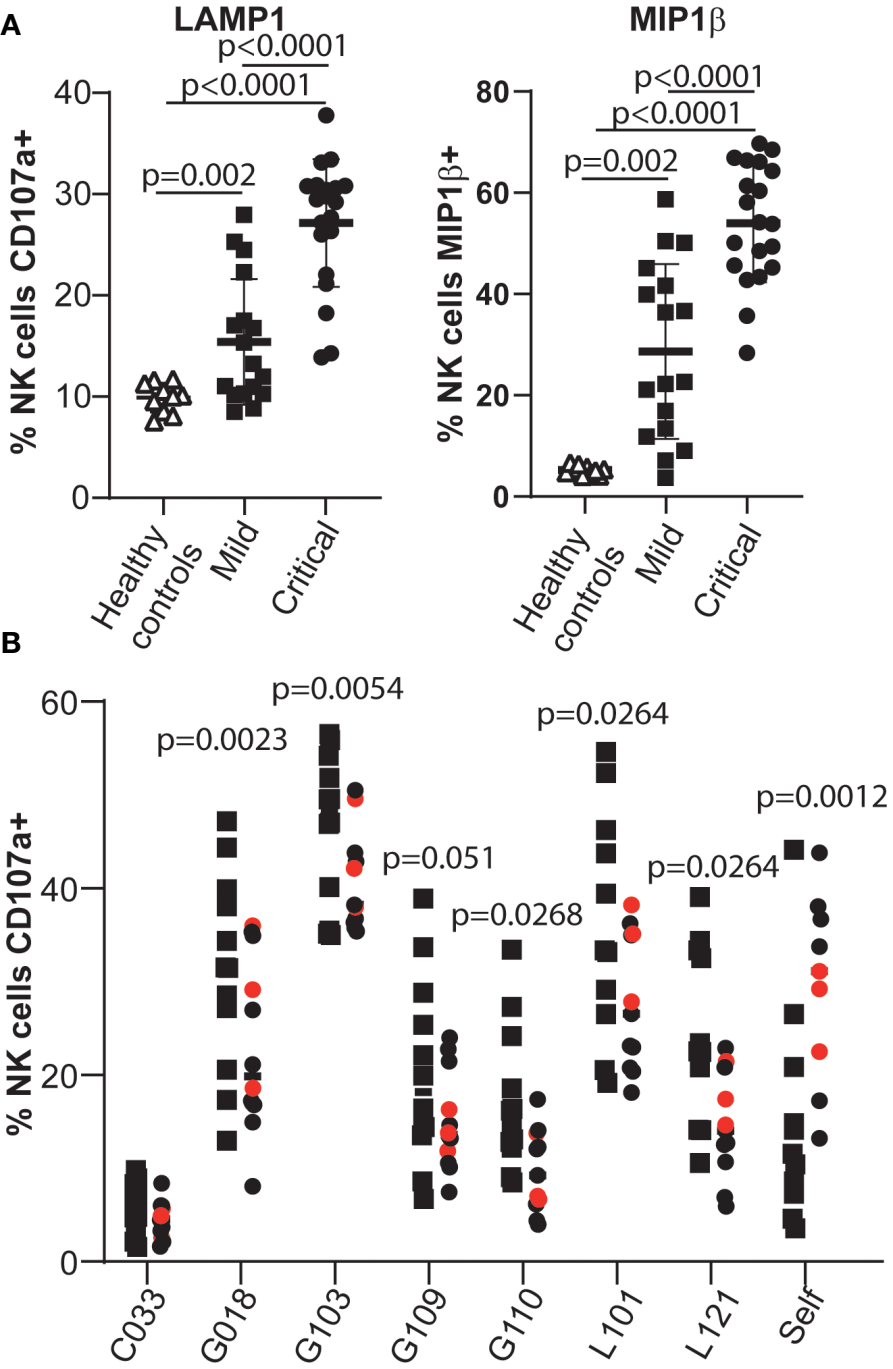
NK cells from patients critically ill with COVID-19 disease are less effective mediators of ADCC than NK cells from patients with mild disease. **(A)** Serum samples from healthy individuals collected pre-pandemia, SARS-CoV-2 infected individuals with mild disease and SARS-CoV-2 infected individuals with life-threatening disease were tested in ADCC assays using PBMC samples from healthy controls. The NK cell response was assayed by measuring degranulation and MIP1β production. **(B)** The ADCC response mediated by NK cells in PBMCs obtained from mild/asymptomatic cases (squares) or from critically ill patients (circles) was assayed using selected serum samples (see **Supplementary Figure 1**). Samples from patients who died due to COVID-19 disease are coloured red. Statistical significance was assessed using two-tailed Mann-Whitney tests and p values are indicated.

Having established sensitive assays to quantify the virus-specific antibody response that develops after infection with SARS-CoV-2 (40, 47), we analysed the function of antibodies in patient serum by assaying whether they could stimulate antibody-dependentNK cell activation in an ELISA-like assay that has previously been used to study antibody-Fc/Fc receptor interactions in the context of humoral immunity to SARS-CoV-2 (48). In initial experiments, we could confirm previous data (48) showing that plasma from patients suffering severe disease contained higher levels of anti-viral antibodies that stimulated NK cell degranulation and cytokine production when PBMCs from healthy donors were used as a source of NK cells (**Figure 1A**). However, little work has been done studying the ADCC capacity of COVID-19 patient NK cells, so, to extend these studies, we assessed the ability of NK cells from patients with either mild or severe disease to respond to anti-viral antibodies recognising the Spike glycoprotein.

For these experiments we compared the phenotype and function of NK cells from two groups of SARS-CoV-2 infected individuals carefully matched for age and sex (**Supplementary Tables 1**, **2**): individuals who presented with mild symptoms of COVID-19 and tested positive in PCR, but recovered spontaneously without hospitalisation, and patients with life-threatening COVID-19 disease that required intensive care and mechanically assisted ventilation. Plasma samples from 6 independent COVID-19 convalescent patients, with different titres of anti-Spike antibodies (**Supplementary Figure 1**), were used as sources of anti-SARS-CoV-2 antibodies.

These data revealed that peripheral blood NK cells from critically ill COVID-19 patients were significantly less potent effector cells than those of patients who developed only very mild signs of disease (**Figure 1B** and **Supplementary Figure 2**). This observation was surprising since previous studies had reported that adaptive NK cells, a population of NK cells generally regarded as being specialised for ADCC, were enriched in the peripheral blood NK cells of COVID-19 patients (31–33, 49), and displayed signs of proliferation and activation (32, 49). However these data are in line with other reports, and our own unpublished observations, documenting that NK cells from severely ill patients mediate lower levels of natural cytotoxicity (31, 33, 38, 50–52).

**Figure 2:**
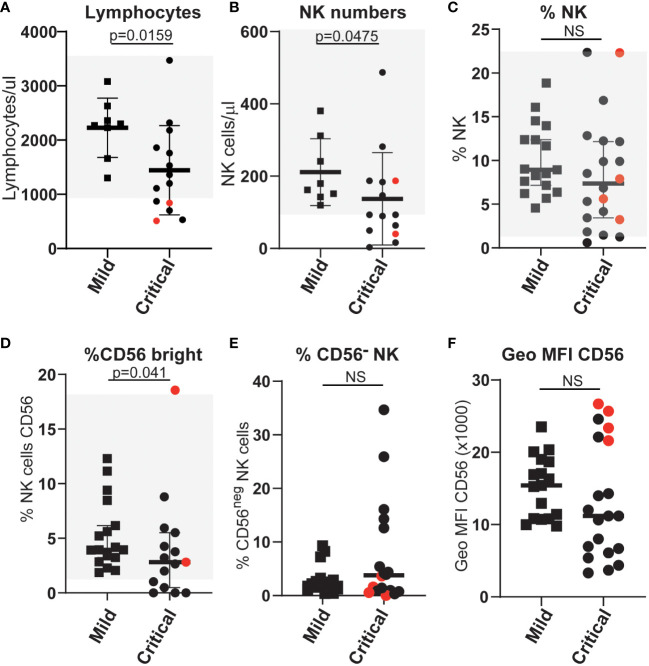
Differences in the numbers and proportions of NK cells **(A–C)** and various NK cell subpopulations **(D–F)** between patients with severe or mild COVID-19 disease. Absolute numbers and proportions of selected NK and NK-cell subsets were measured from COVID-19 patients with either mild or life-threatening disease. Counts were based on full blood count analysis to obtain lymphocyte numbers and flow cytometric analysis to identify population frequencies and fluorescence intensities of staining. Normal ranges, based on analysis of more than 40 anonymous healthy controls, are indicated by the light grey shading. ns: p-value > 0.05.

To search for the basis of this difference in NK cell function we carried out in-depth flow cytometry analyses to further compare NK cells in mildly and severely affected COVID-19 patients. In agreement with multiple other studies, SARS-CoV-2 infected patients, who developed life-threatening disease had significantly lower numbers of NK cells than those individuals with only mild symptoms (**Figures 2A–C**). Moreover, the frequency of CD56^bright^ NK cells, that are generally thought of as less cytotoxic and more potent producers of cytokines than CD56^dim^ NK cells, was significantly reduced in patients with severe disease (**Figure 2D**), whereas this was not the case for patients with mild disease. This observation extends a previous report of a reduction in this population in COVID-19 patients compared to healthy controls (31). However, unlike that study, in this cohort the frequency of CD56^neg^ NK cells (defined by us as CD3^neg^, CD56^neg^, NKp46^pos^) in mild or severely ill patients did not differ significantly (**Figures 2E, F**). Thus although CD56^neg^ NK cells may accumulate in other viral infections, they do not seem to be a useful feature to analyse the evolution of COVID-19 disease.

**Figure 3:**
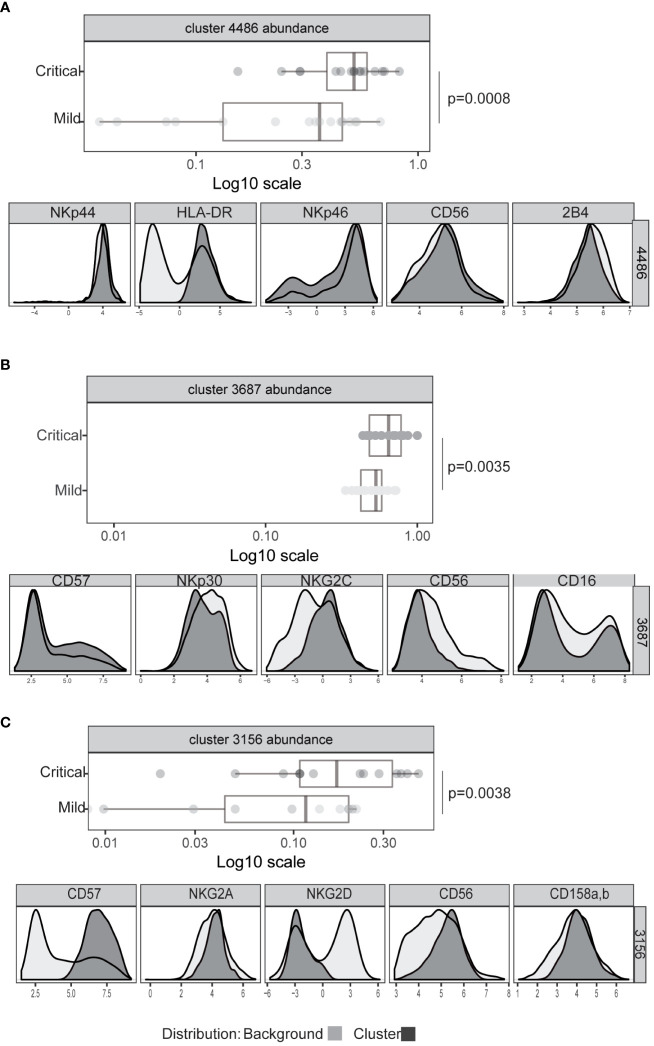
Significant differences in NK cell phenotype between patients with severe or mild COVID-19 disease. Expression intensity of markers that distinguish bulk CD56dim CD16+ NK cells (black line, light grey shaded background) from the cells identified in clusters 4486 **(A)**, 3687 **(B)**, or 3156 **(C)** (dark grey shading). x-axis data represent marker expression intensity; y-axis data represent frequency. The frequency of cells within clusters identified by CITRUS to differentiate COVID-19 patients with mild (light grey) or severe disease (dark grey) subject groups are also shown. Each point represents an individual subject, and horizontal lines represent the group median. Significance was calculated using the Kruskal Wallis test. P values were further adjusted by multiplying by the number of comparisons made (Bonferroni correction).

To rigorously define features of NK cell populations that differ significantly between mildly affected COVID-19 patients and those with life-threatening disease we performed an automated, unbiased comparison of NK cells in these two patient groups using the CITRUS algorhithm (44). These analyses confirmed previous observations of increased HLA-DR expression by NK cells, indicating an increased number of activated NK cells (**Figure 3A**) and increased frequencies of CD57^hi^, CD56^dim^, NKG2C^pos^ (adaptive) NK cells (**Figure 3B**, **Supplementary Figure 3**) in critically ill patients (31–33). However, neither the proportion of CD16+ NK cells nor the levels of CD16 expression by NK cells from these patient groups differed significantly (**Supplementary Figure 4**). This analysis also revealed that a significant difference between critically ill COVID-19 patients and those with only mild disease was the presence of a population of NK cells with markedly reduced expression of the activating receptor NKG2D (**Figure 3C**, **Supplementary Figure 5A**). No significant differences in expression of other receptors such as the KIR2D or NKG2A inhibitory receptors were noted between patients with mild or severe disease (**Supplementary Figure 5B**).

**Figure 4:**
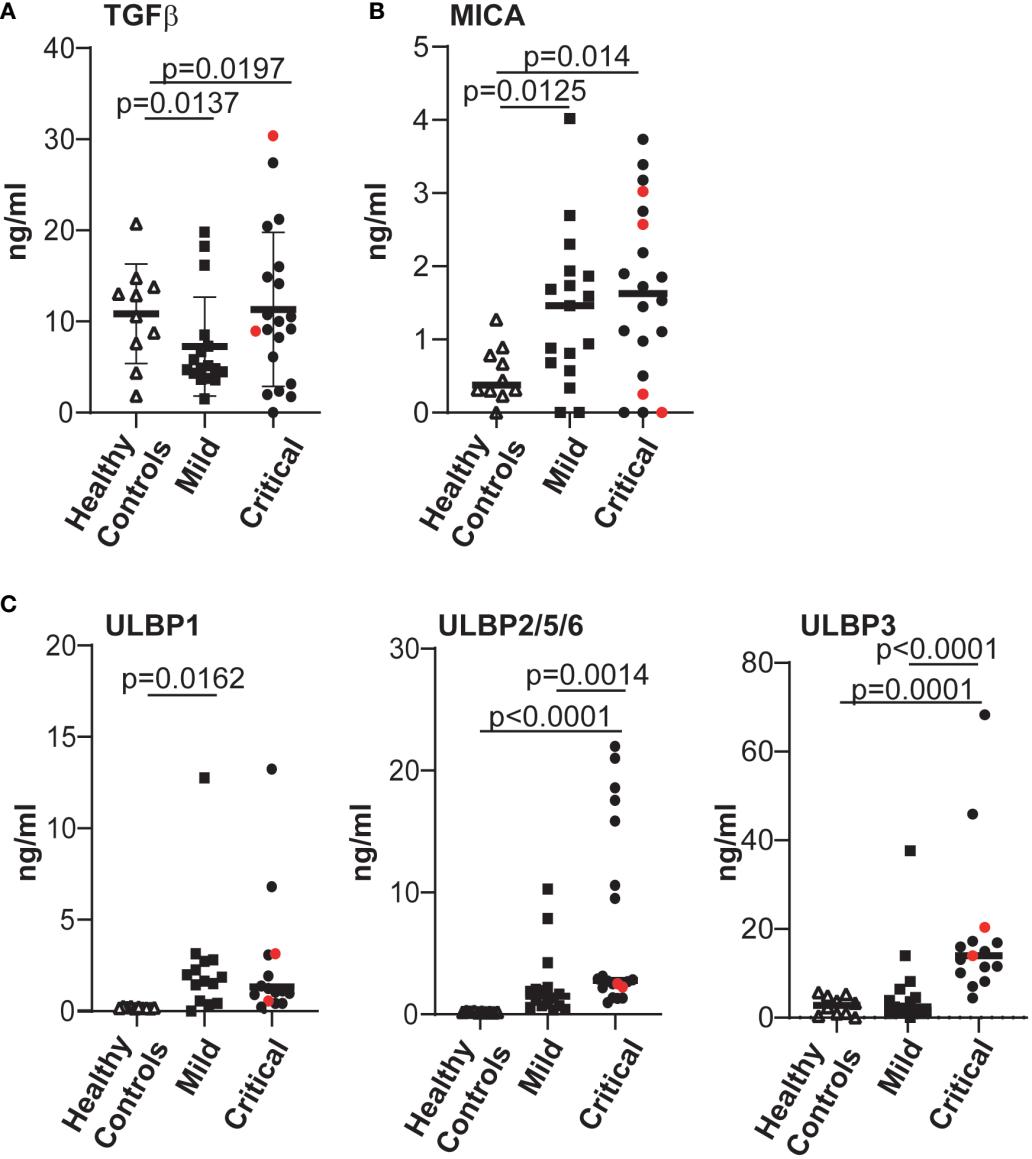
The levels of various factors able to modulate expression of the NKG2D receptor were evaluated and significantly elevated levels of cell-free NKG2D ligands ULBP2 and ULBP3 were found in COVID-19 patients with severe disease. The levels of TGFβ **(A)**, MICA **(B)**; ULBP1, ULBP2/5/6 and ULBP3 **(C)**, all of which can downregulate expression of the NKG2D receptor, present in plasma samples from healthy controls and COVID-19 infected individuals were determined by ELISA. Absorbance data were converted to amounts in ng/ml, by reference to standard curves established using recombinant proteins. Statistical significance was assessed using two-tailed Mann-Whitney tests and p values are indicated.

Since NKG2D expression and NK cell function are known to be modulated by the presence of NKG2D ligands released from cells (53–55) as well as cytokines such as TGFβ (56–58), we tested whether the levels of these molecules were augmented in plasma from COVID-19 patients with life-threatening disease. These experiments revealed that, irrespective of disease severity, the plasma of patients with COVID-19 disease contained levels of the cytokine TGFβ and MICA/B that were significantly elevated compared to controls (**Figures 4A, B**). In contrast, the levels of cell free ULBP2/5/6 and, especially the ULBP3, ligands of the NKG2D receptor, were significantly increased in the plasma of patients with severe COVID-19 disease, but not in plasma from patients with mild disease or healthy controls (**Figure 4C**). Since some cancer patients develop high-titre anti-MICA antibodies that antagonize the suppressive effects of sMICA (59, 60), and the generation of autoantibodies occurs in at least some COVID-19 disease pathologies (61, 62) we also assayed whether the levels of soluble MICA detected might be modulated by anti-MICA antibodies in patients with severe disease, but such antibodies were detected in only a few critically-ill patients (**Supplementary Figure 6A**) and no correlation between the levels of MICA-reactive autoantibodies and soluble MICA in plasma was observed (**Supplementary Figure 6B**).

**Figure 5:**
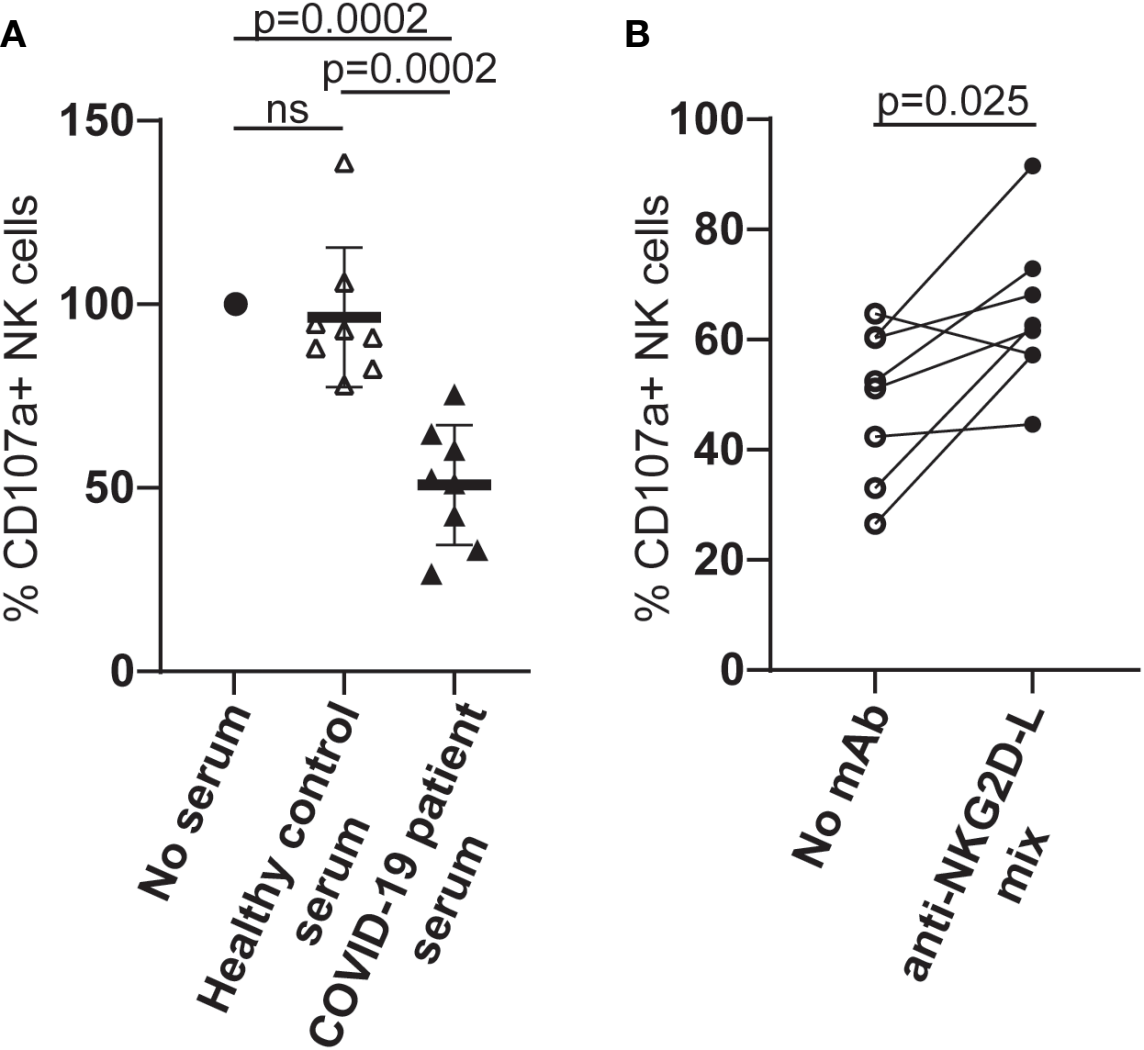
Culture in serum from COVID-19 patients leads to reduced NK cell ADCC activity and this can be partially blocked by NKG2D-L specific mAbs. **(A)** PBMC from healthy individuals were cultured overnight in medium alone, medium supplemented with human serum from healthy controls, or medium with serum from patients who had developed severe COVID-19 disease. NK cell ADCC activity was measured by flow cytometry for LAMP1 expression on CD3-CD56+ NK cells stimulated after exposure to either P815 cells or P815 cells loaded with agonistic CD16-specific mAb (3G8). **(B)** PBMC from healthy donors were cultured in medium to which serum from patients who had developed severe COVID-19 disease, in the presence or absence of a cocktail of MICA/B, ULBP1, ULBP2 and ULBP3-specific mAbs. NK cell ADCC activity was measured as described. Statistical significance was assessed using two-tailed Mann-Whitney tests and p values are indicated. ns: p-value > 0.05.

When PBMCs from healthy individuals were cultured in serum from COVID-19 patients with high levels of cell-free NKG2D-L, the ability of NK cells to degranulate in response to stimulation via CD16A was reduced (**Figure 5A**). These observations are consistent with previous experiments where culture in COVID-19 patient sera was shown to downmodulate NK cell killing of K562 cells (38). To assess the contribution of cell-free NKG2D ligands to the reduced NK cell functionality observed in our experiments, we next tested whether the addition of mAbs to NKG2D-ligands led to any recovery in NK cell activation and observed a partial, but significant, recovery of CD16-stimulated degranulation (**Figure 5B**). The observation that blocking interactions between serum NKG2D-L and the NKG2D receptor increased NK cell ADCC strongly suggests that the presence of cell free NKG2D-L in COVID-19 patient sera contributes to NK cell dysfunction in these patients.

**Figure 6:**
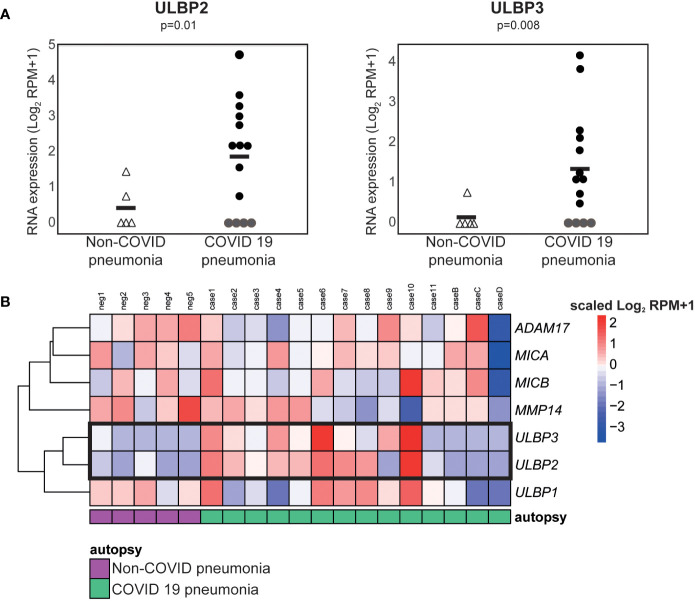
ULBP2 and ULBP3 expression is specifically upregulated in lung tissue from COVID-19 patients. The expression of different NKG2D ligands, and metalloproteases known to be involved in NKG2D-ligand shedding, was analysed using publicly available, pre-processed RNA-seq data obtained on analysis of autopsy specimens from patients who died due after developing COVID-19, or non-COVID, pneumonia. SARS-CoV-2 infected and non-infected lungs were compared using the Log2 normalised counts (plus pseudocount). **(A)** Expression of ULBP2 and ULBP3 mRNAs in these data. **(B)** A heatmap to show expression of various NKG2D ligands and metalloproteases in these samples.

To explore the hypothesis that the elevated levels of cell free ULBP2/5/6 and ULBP3 in patient plasma correlated with increased expression of these molecules in tissues containing SARS-CoV-2 infected cells, we used publicly available RNA-Seq data to compare gene expression in lung samples from patients who had died from COVID-related or non-COVID pneumonia (46). These analyses revealed that SARS-CoV-2 infection was associated with significant increases in levels of mRNA for the NKG2D ligands, ULBP2 and ULBP3 (**Figure 6**) in COVID-19 patients with severe disease.

## Discussion

Although several factors influence the clinical evolution of patients infected with SARS-CoV-2, it is now clear that an inappropriate immune response is a key factor underlying the development of mild or severe disease. Multiple aspects of immunity contribute to this skewing of the balance between immune-mediated virus clearance and immunopathogenicity and here we have compared NK cell phenotype and function in patients with mild/asymptomatic COVID 19 and those with life-threatening disease. The key finding of this work is that the major differences in the NK compartment between patients with mild or life-threatening disease are reduced numbers of NK cells that mediate reduced NK function. Previous work has shown that NK cells from patients with severe COVID-19 disease mediate lower cytotoxic responses against K562 target cells than NK cells from healthy individuals (38, 51), and our data extend those observations to show that NK cells from patients with life threatening disease have a reduced capacity to mediate ADCC.

Mechanistically, the impairment in mediating ADCC does not seem to be simply due to changes in CD16, since neither the frequency nor the intensity of CD16 expression by NK cells varied significantly between the groups of patients with either mild or severe disease (**Supplementary Figure 4**). However, unbiased analyses to compare the phenotype of NK cells in these two cohorts did reveal that NKG2D expression was reduced on NK cells from critically ill patients. TGFβ is known to inhibit the expression of the NKG2D receptor (56) and untimely production of TGFβ that can inhibit NK cell function is a hallmark of severe COVID-19 disease (38). We could confirm that higher levels of this cytokine were present in plasma from COVID-19 patients than from healthy individuals. However, at least in the cohorts we studied, plasma TGFβ levels did not differ significantly between patients with severe or mild COVID-19 disease, perhaps because of when the samples were collected (38). In contrast, significantly elevated levels of cell free ULBP2/5/6 and ULBP3 were found in the serum of critically ill patients compared to outpatients, and exposure to cell-free NKG2D ligands (53, 55), or chronic stimulation via NKG2D (63, 64) are known to trigger reduced NKG2D expression and compromised NK cell function. A prior study hypothesised that NKG2D downregulation on activated NK cells from patients with severe COVID-19 disease might be a consequence of cell-cell interactions since circulating monocytes of hospitalized COVID-19 patients expressed more ULBP-1 and -2/5/6 (ULBP3 expression was not studied) than healthy controls (65). However, in our hands, simply culture in patient sera containing cell free NKG2DL was sufficient to trigger a reduction in ADCC function by healthy donor NK cells that was reversed, at least partially, when blocking NKG2DL-specific mAbs were added to the cultures. These data strongly suggest that the presence of cell-free NKG2DL in blood contributes to the reduced NK function, including ADCC, of patients with severe COVID-19.

In contrast, neither the number of T cells expressing NKG2D nor the intensity of expression varied significantly between patients with severe or mild disease (**Supplementary Figure 7**). It is not clear why NK cells are more susceptible to the effects of cell-free NKG2D ligands than T cells, nevertheless these data are consistent with previous observations where constitutive Rae-1ϵ transgene expression triggered local and systemic NKG2D downregulation with generalised defects in NK cell-mediated cytotoxicity, but only mild CD8+ T cell effects (66). Similarly, whereas incubation with exosomes containing MICA*008 impaired NK cytotoxicity, exposure of CTL to the same numbers of exosomes did not significantly affect the ability of the CTL to specifically lyse target cells (55).

It is not clear how signalling via NKG2D can reduce NK cell responses via other receptors such as CD16, however, our data are consistent with prior reports that murine NK cells chronically stimulated via NKG2D had reduced NKG2D expression and were defective for CD16-mediated ADCC, even though they had normal amounts of the CD16 signalling adaptor molecule FcϵR1γ (64).

It is interesting to observe that elevated levels of cell-free ULBP2/5/6 and ULBP3 found in plasma from critically ill patients is paralleled by increased expression of these ligands in the lungs of patients with severe disease. Infection with a range of pathogens is known to induce expression of ligands for the NKG2D receptor (67), however the mechanisms of ligand release are not yet clear. mRNA expression for a number of proteins known to be involved in shedding of NKG2D ligands did not reveal consistent changes, but this is not unexpected since the activity of many of these enzymes is regulated post-translationally and we do not have access to samples of lung tissue to assess the degree of processing of *e.g.* ADAM17 proprotein or enzyme activity *in vivo*. It is, however, interesting to note that the removal of the pro-domain of ADAMs depends on processing by Furin-like proteases (68) that also cleave the Spike glycoprotein of SARS-CoV-2 to modulate virus entry (69).

In any event, these data suggest that assay of the levels of soluble NKG2D ligands in plasma may have some value as a biomarker for severe disease, although prospective studies will be necessary to properly define their prognostic value.

One limitation of our experiments is that we only have access to plasma and peripheral blood NK cells that may, or may not, have trafficked through sites of SARS-CoV-2 infection. However, our analyses of gene expression data confirm that increased expression of the ULBP2 and ULBP3 ligands is a feature of severe SARS-CoV-2 infection *in vivo*. In this context it is also important to note that although SARS-CoV-2 infection of a lung tumour cell line *in vitro* has been reported to lead to reduced expression of ULBP1 and ULBP2/5/6 (ULBP3 was not studied) (70) (71), it is not clear how representative these studies are of what might occur in the much more complex *in vivo* environment of virus-infected lung tissue infiltrated by multiple immune effector cells producing a wide range of cytokines. Finally, it will be important in the future to carry out similar analyses from patients affected by other respiratory virus infections in order to assess the specificity of the different features of these responses for SARS-CoV-2 infection.

## Data availability statement

The original contributions presented in the study are included in the article/**Supplementary Material**. Further inquiries can be directed to the corresponding author.

## Ethics statement

Samples used in this study were provided by the Biobank Hospital Universitario Puerta de Hierro Majadahonda (HUPHM)/Instituto de Investigación Sanitaria Puerta de Hierro-Segovia de Arana (IDIPHISA) (PT17/0015/0020 in the Spanish National Biobanks Network). The use of these samples was approved by the Research Ethics Committee of the CSIC (173/2020) and the biobank (AC0099-A-2021). The studies were conducted in accordance with the local legislation and institutional requirements. Written informed consent for participation was not required from the participants or the participants’ legal guardians/next of kin because anonymized human samples used came from Biobank.

## Author contributions

HR: Conceptualization, Funding acquisition, Project administration, Supervision, Writing – original draft, Writing – review & editing. DF: Investigation, Formal analysis, Writing – review & editing. ÁG: Formal analysis, Investigation, Writing – review & editing. JC: Resources, Writing – review & editing. MV: Conceptualization, Funding acquisition, Resources, Supervision, Writing – review & editing.
