# Supplementary material for: Elevated levels of cell-free NKG2D-ligands modulate NKG2D surface expression and compromise NK cell function in severe COVID-19 disease
Source: Front Immunol. 2024 Feb 12;15:1273942. doi: 10.3389/fimmu.2024.1273942 (PMC10895954; doi:10.3389/fimmu.2024.1273942)
Supplement: Supplementary file 1 [file DataSheet_1.pdf]

Supplementary Table 1

	Days between diagnosis and collection of PBMCs			
	**<20**	**20-40**	**40-60**	**>60**
***Critical*[a]**	4	7*	5*	3
***Outpatient*[b]**	2	7	9	3

[a] Admitted to intensive care and required mechanical ventilation

[b] Outpatient care only

* 4 deaths in ICU in group of critically patients (♂, 63 and 71 years of age; ♀, 51 and 72 years of age)

Supplementary Table 2

	Average Age (range)		*Total numbers*	
	Men	Women	Men	Women
Critical	60* (47 – 76)	56* (46, 51, 72)	17	3
Outpatient	53 (42 – 80)	58 (56, 57, 61)	13	3
Pre-COVID controls	56 (42 – 68)	54 (46, 51, 57, 61)	26	4

4 deaths in group of critically patients (♂, 63 and 71 years of age; ♀, 51 and 72 years of age)

Supplementary Table 3. Antibodies used		
**Antibody**	**Clone**	**Supplier**
Pacific Blue™ anti-human CD3 Antibody	UCHT1	Biolegend
FITC anti-human CD57 Antibody	HNK-1	Biolegend
PE anti-human CD314 (NKG2D)	1D11	Biolegend
PE/Cy7 anti-human CD16 Antibody	3G8	Biolegend
APC anti-human CD337 (NKp30)	P30-15	Biolegend
PE/Cy7 anti-human CD158 (KIR2DL1/S1/S3/S5)	HP-MA4	Biolegend
PE/Cy7 anti-human CD158b (KIR2DL2/L3, NKAT2)	DX27	Biolegend
APC anti-human CD336 (NKp44)	P44-8	Biolegend
PE anti-human CD335 (NKp46)	900	Biolegend
APC/Cyanine7 anti-human HLA-DR Antibody	L243	Biolegend
V500 Mouse Anti-Human CD16	3G8	BD Biosciences
Human NKG2C/CD159c PE-conjugated Antibody	134591	R&D Systems
CD159a-APC	Z199	Beckman Coulter
CD56-PC5	N901	Beckman Coulter
CD107a-APC	H4A3	Biolegend
MIP1β-PE	D21-1351	Beckman Coulter
MICA	mAb13002	R&D Systems
MICA-biotinylated	BAF1300	R&D Systems
ULBP1	MAB1380	R&D Systems
ULBP1	AF1380	R&D Systems
ULBP1-biotinylated	BAF1380	R&D Systems

ULBP2/5/6	MAB1298	R&D Systems
ULBP2/5/6	AF1298	R&D Systems
ULBP2/5/6-biotinylated	BAF1298	R&D Systems
ULBP3	MAB1517	R&D Systems
ULBP3	AF1517	R&D Systems
ULBP3-biotinylated	BAF1517	R&D Systems
