# Supplementary material for: Elevated levels of cell-free NKG2D-ligands modulate NKG2D surface expression and compromise NK cell function in severe COVID-19 disease
Source: Front Immunol. 2024 Feb 12;15:1273942. doi: 10.3389/fimmu.2024.1273942 (PMC10895954; doi:10.3389/fimmu.2024.1273942)
Supplement: Supplementary file 2 [file DataSheet_2.pdf]

Supplementary Figure 1

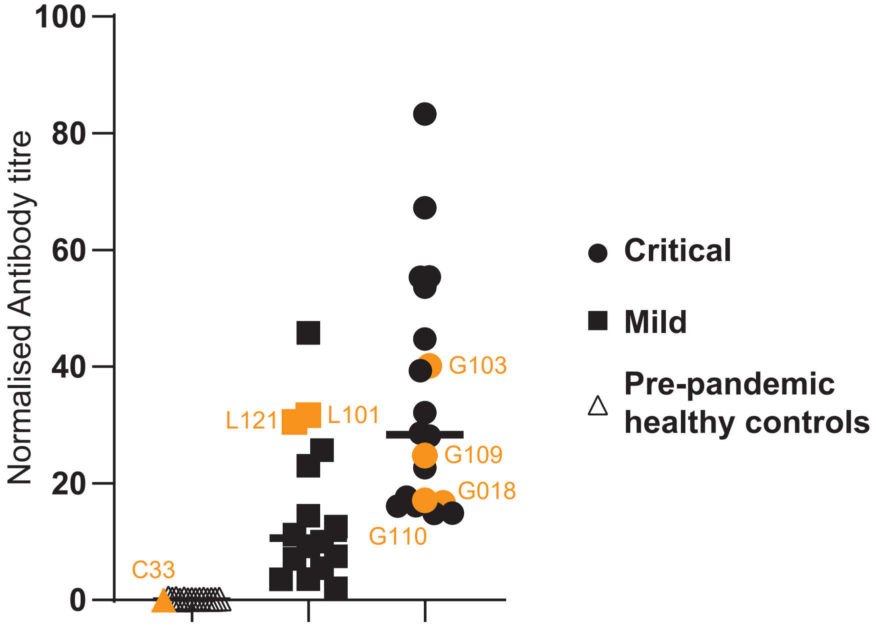

Supplementary Figure 1
The titres of SARS CoV 2 specific antibodies present in serum samples from the infected individuals analysed in this paper were determined as previously described (36).
