# Supplementary figures and images for: Elevated levels of cell-free NKG2D-ligands modulate NKG2D surface expression and compromise NK cell function in severe COVID-19 disease
Source: Front Immunol. 2024 Feb 12;15:1273942. doi: 10.3389/fimmu.2024.1273942 (PMC10895954; doi:10.3389/fimmu.2024.1273942)

Supplementary Figure 2

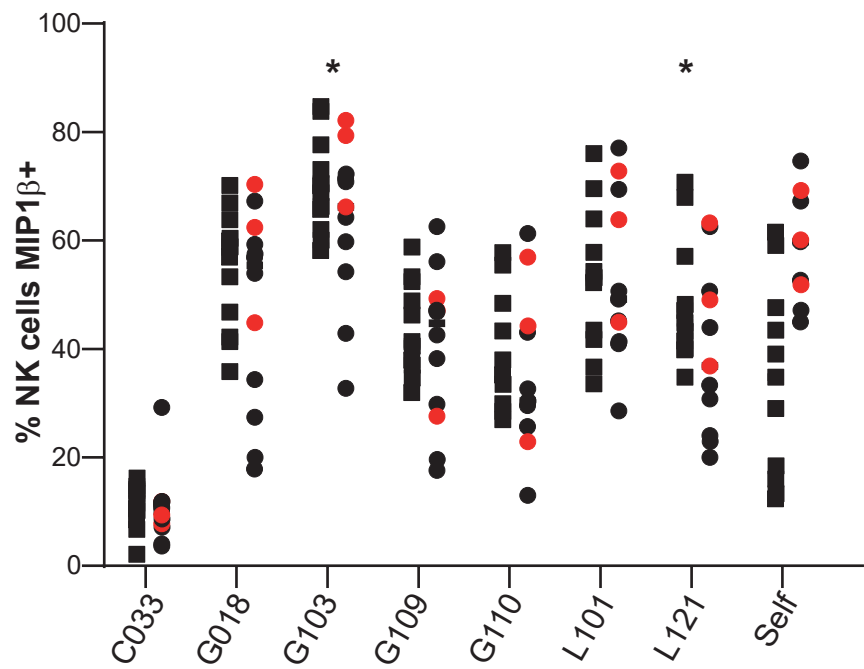

Supplement: Supplementary file 3 [file DataSheet_3.pdf]
