# Supplementary material for: Elevated levels of cell-free NKG2D-ligands modulate NKG2D surface expression and compromise NK cell function in severe COVID-19 disease
Source: Front Immunol. 2024 Feb 12;15:1273942. doi: 10.3389/fimmu.2024.1273942 (PMC10895954; doi:10.3389/fimmu.2024.1273942)
Supplement: Supplementary file 4 [file DataSheet_4.pdf]

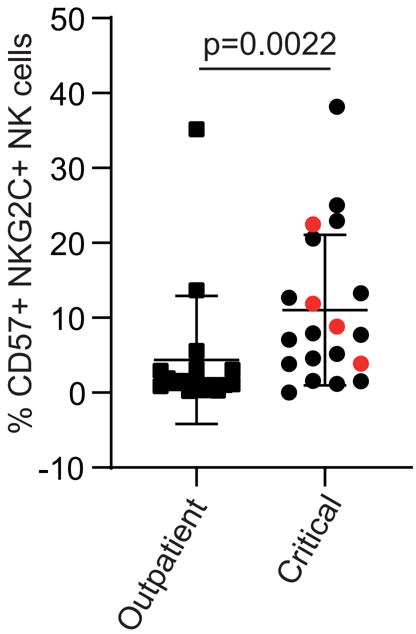

Supplementary Figure 3
PBMCs were thawed in warmed RPMI medium and incubated overnight at 37°C 5% CO_2. The next day cells were counted and some were stained for phenotyping by flow cytometry while the rest were used in functional assays.
