# Supplementary material for: Elevated levels of cell-free NKG2D-ligands modulate NKG2D surface expression and compromise NK cell function in severe COVID-19 disease
Source: Front Immunol. 2024 Feb 12;15:1273942. doi: 10.3389/fimmu.2024.1273942 (PMC10895954; doi:10.3389/fimmu.2024.1273942)
Supplement: Supplementary file 6 [file DataSheet_6.pdf]

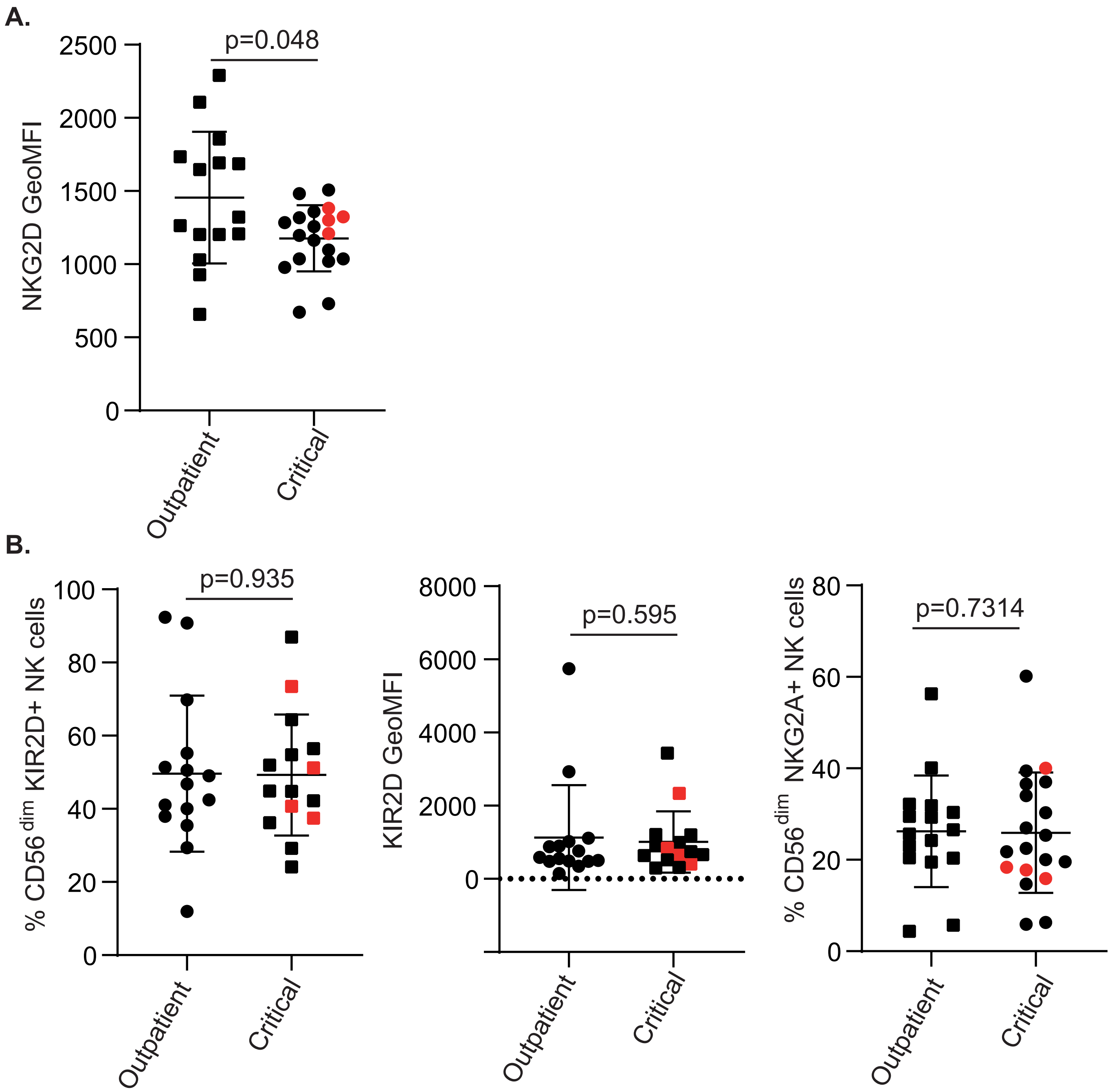

Supplementary Figure 5

Thawed PBMCs were stained with CD3 and CD56 mAbs to identify NK cells and then the fraction of NK cells expressing the indicated markers and the levels of expression of those markers were determined by staining with specific mAb (Supplementary Table 3) and flow cytometry analysis.
