# Supplementary material for: Elevated levels of cell-free NKG2D-ligands modulate NKG2D surface expression and compromise NK cell function in severe COVID-19 disease
Source: Front Immunol. 2024 Feb 12;15:1273942. doi: 10.3389/fimmu.2024.1273942 (PMC10895954; doi:10.3389/fimmu.2024.1273942)
Supplement: Supplementary file 7 [file DataSheet_7.pdf]

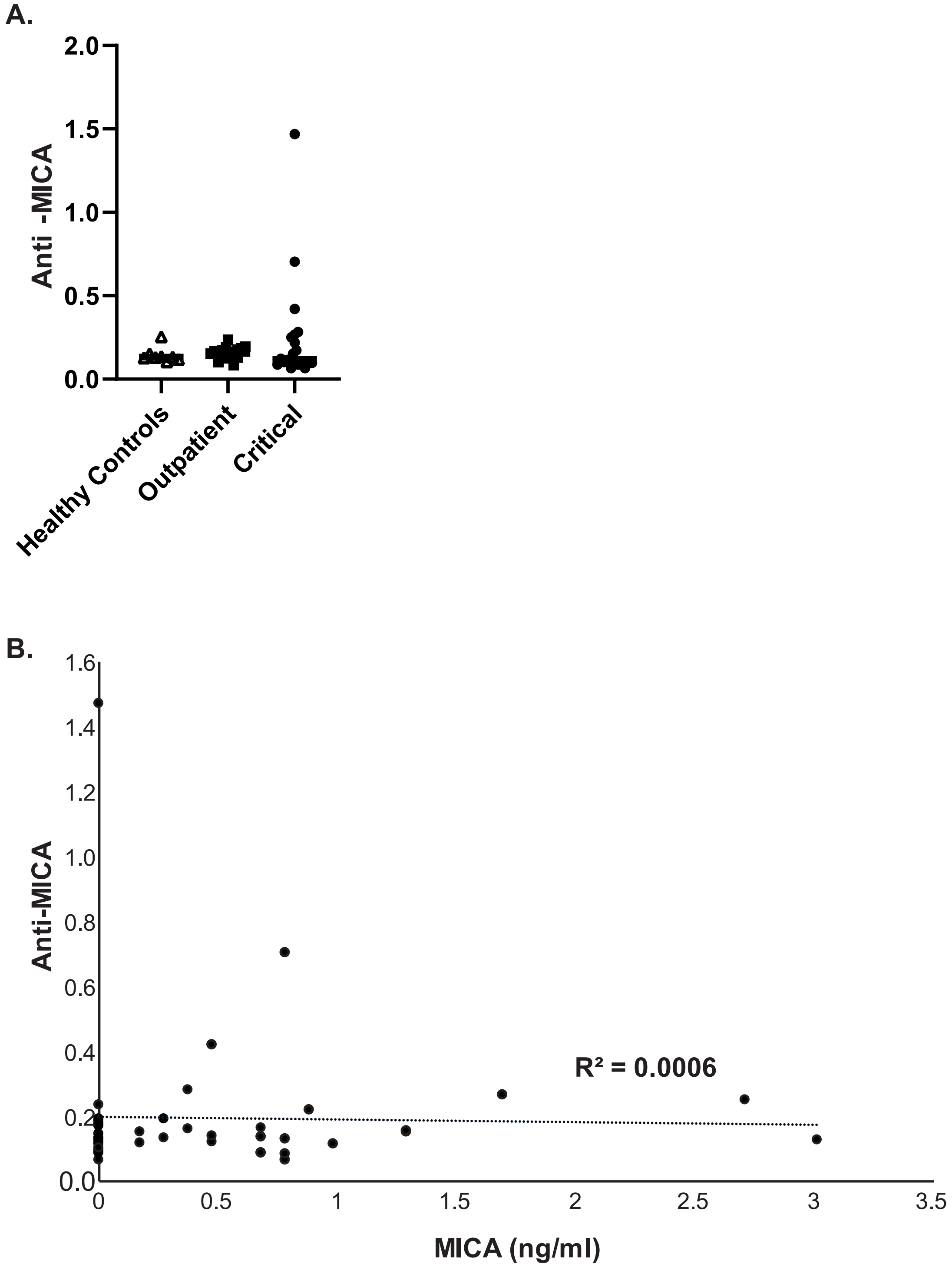

Supplementary Figure 6

A. The levels of anti-MICA antibodies present in patient sera were determined by ELISA against immobilised recombinant MICA protein. B. The levels of MICA-specific antibodies and soluble MICA in patient sera were determined by ELISA.
