# Supplementary material for: Elevated levels of cell-free NKG2D-ligands modulate NKG2D surface expression and compromise NK cell function in severe COVID-19 disease
Source: Front Immunol. 2024 Feb 12;15:1273942. doi: 10.3389/fimmu.2024.1273942 (PMC10895954; doi:10.3389/fimmu.2024.1273942)
Supplement: Supplementary file 8 [file DataSheet_8.pdf]

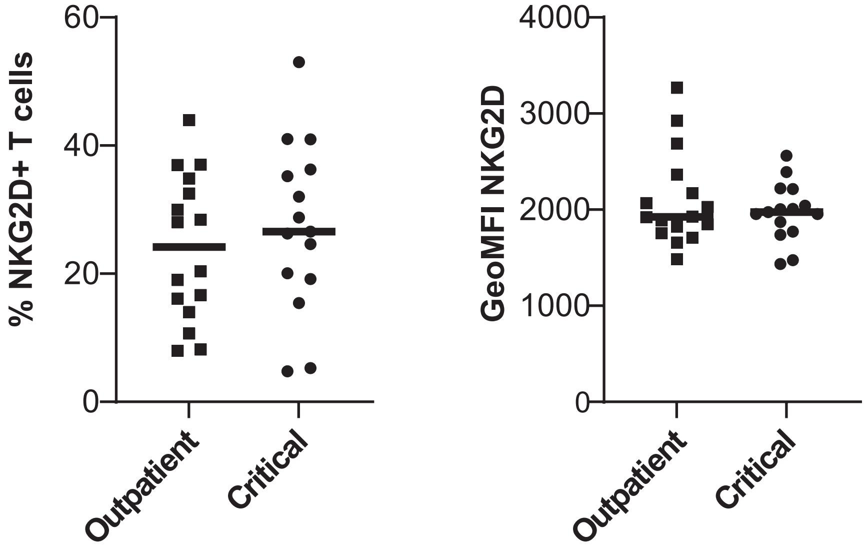

Supplementary Figure 7
Thawed PBMCs were stained with a CD3 specific mAb to identify T cells and then the fraction of T cells expressing the NKG2D receptor and the levels of expression of NKG2D were determined by flow cytometry analysis using the 1D11 mAb (Supplementary Table 3).
